# Ferumoxytol across the age spectrum: a single center experience of safety

**DOI:** 10.1186/1532-429X-18-S1-P259

**Published:** 2016-01-27

**Authors:** Kim-Lien Nguyen, Takegawa Yoshida, Ihab Ayad, Brian Reemtsen, Gary M Satou, Peng Hu, Isidro Salusky, J Paul Finn

**Affiliations:** 1 Department of Radiological Sciences, David Geffen School of Medicine at UCLA, Los Angeles, CA USA; 2 Department of Anesthesiology, David Geffen School of Medicine at UCLA, Los Angeles, CA USA; 3 Department of Cardiothoracic Surgery, David Geffen School of Medicine at UCLA, Los Angeles, CA USA; 4 Division of Pediatric Cardiology, David Geffen School of Medicine at UCLA, Los Angeles, CA USA; 5 Division of Cardiology, David Geffen School of Medicine at UCLA and VA Greater Los Angeles Healthcare System, Los Angeles, CA USA; 6 Division of Pediatric Nephrology, David Geffen School of Medicine at UCLA, Los Angeles, CA USA

## Background

Ferumoxytol is used for parenteral iron therapy in patients with chronic kidney disease (CKD). Because of its uniquely powerful properties as an intravascular MR contrast agent, there is growing interest in the safety of ferumoxytol as a possible alternative to gadolinium-based contrast agents in patients with CKD and in patients with congenital heart disease. We reviewed the frequency, type and severity of adverse reactions to ferumoxytol as an MRI contrast agent over a broad spectrum of ages and indications in a single center study.

## Methods

Following informed consent and with approval from our IRB, we performed ferumoxytol enhanced MRA (FEMRA) for the assessment of pathologic arterial and /or venous anatomy in 165 patients (age 36 ± 28 years, range 3 days to 94 years, 38% female). Both bolus and slow infusions were given (total dose of 4 mg /kg, 166 injections). Sixty-five patients were examined under general anesthesia; three had pacemakers and six were pregnant. First pass and steady state FEMRA were performed in 119 patients and 46 patients had only steady state imaging. Continuous monitoring of ECG, pulse oximetry, and non-invasive blood pressure was performed in all patients and the electronic medical records were reviewed to assess for follow up events.

## Results

In all cases, patients remained stable throughout the FEMRA studies and there were no serious adverse events. In two patients, systolic blood pressure (SBP) transiently decreased by 10-15 mmHg and in 22 patients SBP increased by 10-15 mmHg. In eight patients with congenital heart disease, blood oxygenation decreased by 1-5% during the MRI study. Three patients developed nausea following injection of ferumoxytol but in all cases the studies were completed successfully.

## Conclusions

In our single center experience, there were no serious adverse events with the use of ferumoxytol for MRI, whether injected as a bolus or infused slowly. Although encouraging, more patient studies from multiple centers will be needed fully to define the safety profile of ferumoxytol for diagnostic use.

**Figure 1:**
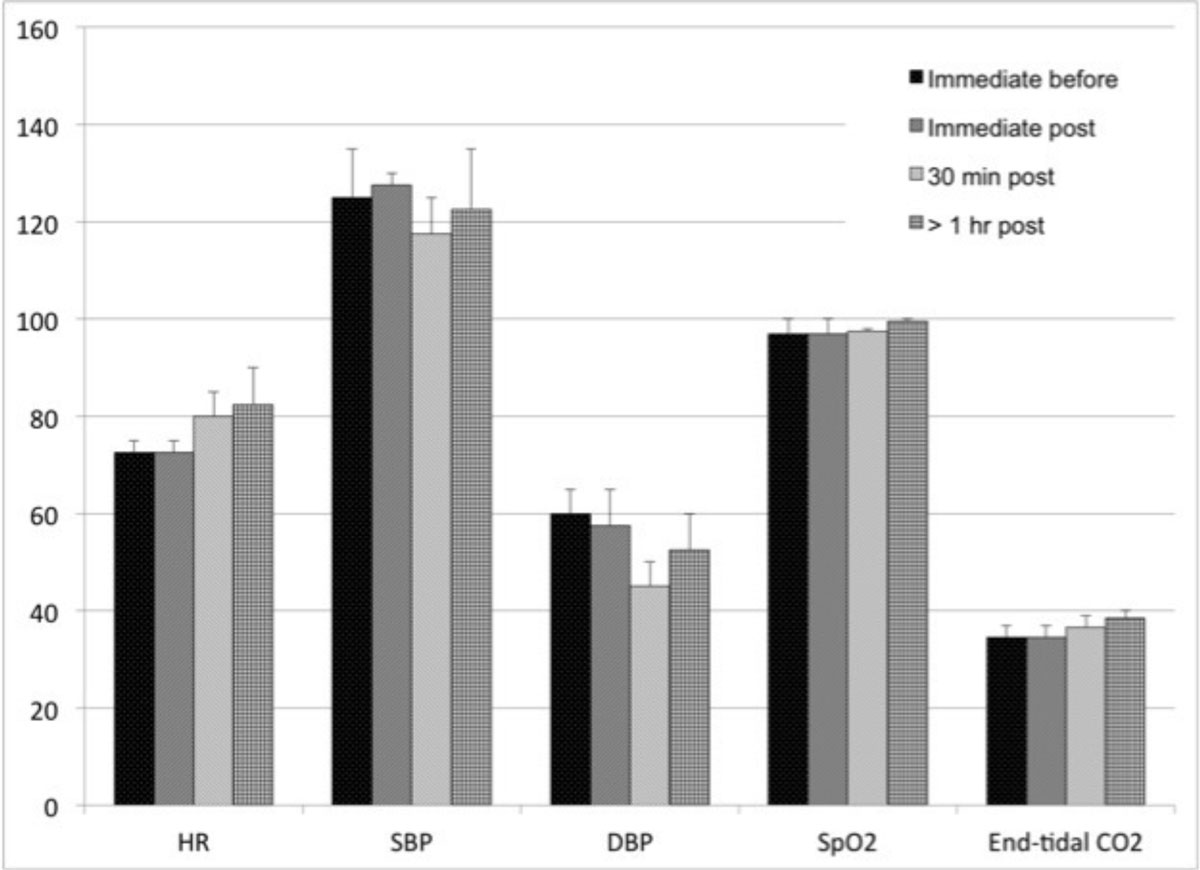
**Patients with continuous monitoring under anesthesia**. Heart rate (HR), systolic (S) and diastolic (D) blood pressure (BP), pulse oximetry, and end-tidal CO2 immediately before ferumoxytol injection, immediately post, 30 minutes post, and >1 hour post injection. Units are BP=mmHg; End-tidal CO2=mmHg, HR=beats per min, spO2=%

